# Effect of Periodontitis and Scaling and Root Planing on Risk of Pharyngeal Cancer: A Nested Case—Control Study

**DOI:** 10.3390/ijerph18010008

**Published:** 2020-12-22

**Authors:** Ping-Ju Chen, Yin-Yang Chen, Chiao-Wen Lin, Ying-Tung Yeh, Han-Wei Yeh, Jing-Yang Huang, Shun-Fa Yang, Chao-Bin Yeh

**Affiliations:** 1 Institute of Medicine, Chung Shan Medical University, Taichung 402, Taiwan; 139370@cch.org.tw (P.-J.C.); jeff80329@hotmail.com (Y.-Y.C.); wchinyang@gmail.com (J.-Y.H.); 2 Department of Dentistry, Changhua Christian Hospital, Changhua 500, Taiwan; 3 Department of Surgery, Chung Shan Medical University Hospital, Taichung 402, Taiwan; 4 Institute of Oral Sciences, Chung Shan Medical University, Taichung 402, Taiwan; cwlin@csmu.edu.tw; 5 Department of Dentistry, Chung Shan Medical University Hospital, Taichung 402, Taiwan; yehtungtung@hotmail.com; 6 Graduate School of Dentistry, School of Dentistry, Chung Shan Medical University, Taichung 402, Taiwan; 7 School of Medicine, Chang Gung University, Taoyuan City 333, Taiwan; george66889@gmail.com; 8 Department of Medical Research, Chung Shan Medical University Hospital, Taichung 402, Taiwan; 9 Department of Emergency Medicine, School of Medicine, Chung Shan Medical University, Taichung 402, Taiwan; 10 Department of Emergency Medicine, Chung Shan Medical University Hospital, Taichung 402, Taiwan

**Keywords:** periodontitis, pharynx cancer, scaling, root planing

## Abstract

This study investigated the association between periodontitis and the risk of pharyngeal cancer in Taiwan. For this population-based nested case–control study using the Longitudinal Health Insurance Database derived from Taiwan’s National Health Insurance Research Database, we identified patients (*n* = 1292) who were newly diagnosed with pharyngeal cancer between 2005 and 2013 and exactly paired them with propensity score matched control subjects (*n* = 2584). Periodontitis and scaling and root planing (SRP) were identified before the index date. Pharyngeal cancer was subdivided into 3 subgroups on the basis of anatomic location: nasopharyngeal cancer, oropharyngeal cancer, and hypopharyngeal cancer. A multiple conditional logistic regression model was applied to analyze the adjusted odds ratio (aOR). Periodontitis was associated with an increased risk of pharyngeal cancer (aOR, 1.57; 95% confidence interval (CI), 1.17 to 2.10), especially oropharyngeal cancer (aOR, 2.22; 95% CI, 1.07 to 4.60). We found a decreased risk of pharyngeal cancer in patients who had undergone SRP (aOR, 0.77; 95% CI, 0.61 to 0.96). In conclusion, this study showed that periodontitis was associated with an increased risk of pharyngeal cancer and SRP exerted a protective effect against pharyngeal cancer. Our results suggest that treating periodontitis and performing SRP, which are modifiable factors in oral health, in clinical practice may provide an opportunity to decrease the disease burden of pharyngeal cancer in Taiwan.

## 1. Introduction

Head and neck cancer (HNC), including cancers of the oral cavity, pharynx, and larynx, accounts for approximately 600,000 cases worldwide annually [1]. In Taiwan, HNC is one of the leading cancers, with an incidence of 22 per 100,000 and mortality rate of 8.2 per 100,000 for oral and pharyngeal cancers in 2014, and both the incidence and mortality rate are higher among men [2]. Among HNC cases, pharyngeal cancer can be further classified as nasopharyngeal, oropharyngeal, or hypopharyngeal cancer according to anatomic location. The incidence of nasopharyngeal, oropharyngeal, and hypopharyngeal cancers is 9.47, 5.09, and 6.28 per 100,000, respectively.

Major risk factors for HNC have been well studied and established; they include the use of alcohol, tobacco products, and betel nuts. Dhull et al. performed a systematic review and identified the major roles and interactions of alcohol consumption and smoking tobacco products in the development of HNC [3]. Widespread in certain regions of Asia, especially in Taiwan, betel nut chewing is also an independent risk factor for the carcinogenesis of HNC [4,5]. Other nonclassical risk factors include viral infection (i.e., Epstein–Barr virus [6]; human papillomavirus [7]); diet [8]; and systemic diseases, such as diabetes mellitus [9], metabolic syndrome [10], and chronic inflammation [11].

Oral hygiene and periodontal diseases are also currently considered risk factors for HNC. Börnigen et al. found significant changes in oral bacterial composition and function, which are associated with risk of HNC, between patients with HNC and a control group [12]. Guha et al. performed 2 multicentric case–control studies on oral health and risk of HNC and found that periodontal disease and mouthwash use more than twice daily may be independent causes of HNC [13]. Additionally, a recent systematic review showed a positive association between periodontitis and oral cancer [14].

However, most current studies have focused on oral cancer, with only a few studies focusing on the relationship between periodontitis and pharyngeal cancer. Furthermore, most studies have mainly been conducted in the USA or European countries; thus, data and evidence concerning people in Eastern countries, where the prevalence of disease and management of oral health may be different, are lacking. Specifically, no results have been reported in Taiwan on the association of periodontitis with pharyngeal cancer and the impact of periodontal management. Previous studies mainly focused on dental care during and after radiotherapy for head and neck patients. The effect of dental non-surgical and surgical therapy on cancer risk for the general population are less mentioned, while SRP are common non-surgical periodontal therapy, under coverage of National Health Insurance every six months in Taiwan. Hence, the objective of this study was to evaluate periodontitis, SRP and the risk of pharyngeal cancer by performing a nested case–control study based on the Taiwan National Health Insurance Research Database (NHIRD).

## 2. Material and Methods

### 2.1. Data Source

The NHIRD, a population-based insurance claims database, contains data of 98–99% of the Taiwanese population in 2010. There were approximately 27.38 million people in the 2010 Registry for Beneficiaries of the NHIRD. We used the Longitudinal Health Insurance Database (LHID) 2010, a subset of the NHIRD, which includes roughly 1 million beneficiaries who were randomly sampled from the registry file of the NHIRD in 2010. The records of expenditures for inpatient, ambulatory, dental care, and pharmacy services are included in the LHID. For analysis, we selected variables including demographics (birth year, sex, location of registry, and insurance amount), date of visit or admission/discharge, diagnosis (International Classification of Diseases, Ninth Revision, Clinical Modification (ICD-9-CM) codes), prescriptions, and procedures. To protect the subjects’ personal privacy, all identifiers of beneficiaries or health care providers were encrypted before the data were released by the National Health Research Institutes. The study protocol was approved by the Institutional Review Board (IRB) of Chung Shan Medical University Hospital (IRB approval no. CS2-15061).

### 2.2. Study Design and Patient Selection

We conducted the present nested case–control study to evaluate the risk of pharyngeal cancer in patients with periodontitis. Initially, there were 978,918 individuals and 1997–2013 records in the raw datasets. First, we excluded the prevalent cases (*n* = 13,328) with any cancer before 2005; then, we identified the patients (*n* = 1332) who were newly diagnosed with pharyngeal cancer (ICD-9-CM diagnosis codes 146.x–148.x) between 2005 and 2013. The first date of pharyngeal cancer diagnosis was defined as the index date. A previous study evaluated the validity of the diagnosis of cancer between 2001 and 2012 in the NHIRD and found that the positive predictive value was 94% for all cancers and that the positive predictive value was 94.40 and the negative predictive value was 99.99 for oral cavity, oropharyngeal, and hypopharyngeal cancers. In an observational study design, confounding bias is the major problem that results in misleading conclusions. Propensity score matching was used to balance the demographics, comorbidities, length of stay, and time of dental visit at baseline between the case and control groups. The propensity score (the probability of diagnosis with pharyngeal cancer) for each individual was estimated using logistic regression. Furthermore, the greedy nearest neighbor matching algorithm without replacement was used to select 2 optimal control subjects for each subject with pharyngeal cancer at the index date. To avoid control subjects withdrawing before the index date, we also checked the registry status of control subjects, and the matched control subjects who died before the index date were taken back to the control pool, and the other control subjects were resampled until a full sample of matched control subjects was selected.

### 2.3. Management of Periodontitis before Index Date

We identified the diagnosis of periodontitis (ICD-9-CM diagnosis codes 523.3–523.5) between 1 January 1997, and the index date and calculated the periodontitis history as the interval between the date of first diagnosis with periodontitis and the index date. The history of periodontitis was divided into 3 categories based on the diagnosis over the past 6 years: nonperiodontitis, 0 to 3 years, and 3 to 6 years. Scaling and root planing (SRP) treatment was considered to be the moderator on the effect of periodontitis. The total amount of SRP was the most important indicator of management for periodontitis, and we counted the total amount of SRP within 6 years (not including index date) before the index date. The use of SRP is time-dependent, and the binary variable (yes/no) of SRP was identified annually before the index date. Temporary SRP (only used a long time [3 to 6 years before the index date] ago or only used recently (0 to 3 years before the index date) and permanent SRP (identified in both periods before the index date) were compared with regard to the risk of pharyngeal cancer.

### 2.4. Other Factors Associated with Risk of Pharyngeal Cancer

The comorbidities considered as covariates in the analysis were diabetes mellitus (ICD-9-CM code 250), hypertension (ICD-9-CM codes 401–405), hyperlipidemia (ICD-9-CM code 272), chronic kidney disease (ICD-9-CM codes 582, 583, 585, 586, 588), depression (ICD-9-CM code 311), stroke (ICD-9-CM codes 430–436), ulcer of esophagus (ICD-9-CM codes 530.1, 530.2), allergies (ICD-9-CM codes 477, 493, 708, 691, 693.0, 693.1, 955.0, 955.1, 955.3), chronic obstructive pulmonary disease (ICD-9-CM codes 490, 491, 492, 494, 496), and hepatitis (ICD-9-CM codes 070, 571.4, 573.1–573.3). In addition, medical service use, including length of hospital stay (days) and visits for dental service before the index date, might affect the chance of diagnosis of periodontitis, use of SRP, and even the diagnosis of pharyngeal cancer.

### 2.5. Statistical Analysis

We used the chi-square test to evaluate the homogeneity of baseline characteristics between the case and control groups. Conditional logistic regression was conducted to estimate the odds ratio (OR) of pharyngeal cancer. The use of SRP was strongly associated with history of periodontitis; therefore, the effect of SRP on pharyngeal cancer was analyzed, stratified by duration of periodontitis. SAS version 9.4 (SAS Institute, Cary, NC, USA) was used for statistical analysis, and a *p* value less than 0.05 was defined as statistically significant.

## 3. Results

### 3.1. Characteristics of Study Subjects

**Figure 1:**
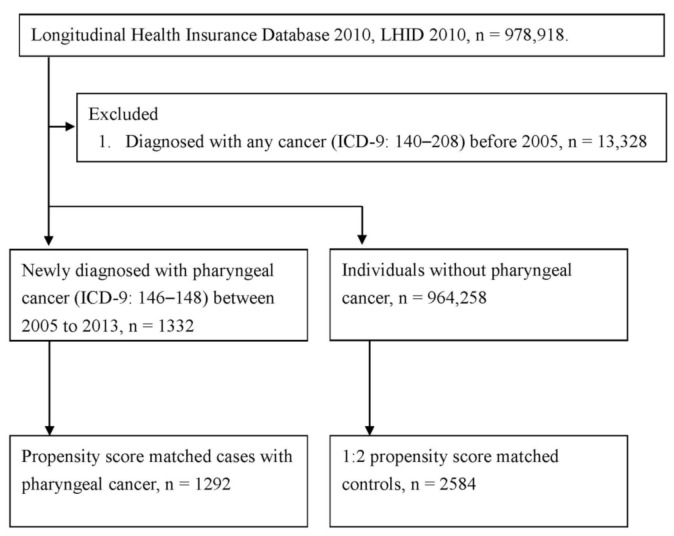
Flow chart for patient’s selection.

We identified 1292 patients newly diagnosed with pharyngeal cancer, including oropharyngeal cancer (*n* = 220), nasopharyngeal cancer (*n* = 845), and hypopharyngeal cancer (*n* = 227), from 2005 to 2013. After 1:2 propensity score matching, 2584 individually matched control subjects were finally included (Figure 1). Among the pharyngeal cancer group, 74.46% were men, and the mean age was 50.80 years (standard deviation: 13.31) at diagnosis with cancer. The characteristics of the patients with pharyngeal cancer and their matched control subjects are shown in Table 1. There was no statistically significant difference in age, sex, urbanization, comorbidities, length of hospital stay, or time of dental visit between the pharyngeal cancer and control groups (*p* > 0.05). Moreover, the characteristics of the subset analysis of oropharyngeal, nasopharyngeal, and hypopharyngeal cancer are presented in Table 1, and the differences remained not significantly different (*p* > 0.05) after individual matching.

### 3.2. Effect of Periodontitis or SRP on the Risk of Pharyngeal Cancer

**Figure 2:**
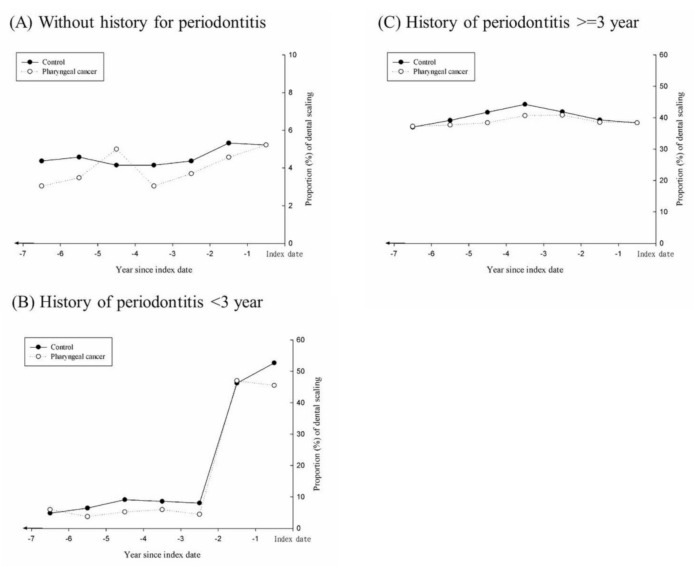
The annual proportion (%) of scaling and root planing in both groups within 6 years before index date.

Figure 2 shows the annual proportion of subjects who received SRP in both groups. After stratification according to history of periodontitis, Figure 2A–C show that the case group had a lower annual proportion of SRP use. Especially in the subset with a history of periodontitis, the case group had a lower proportion of SRP during 2 to 6 years before the index date.

In Table 2, the results show the effect of periodontitis or SRP on the risk of pharyngeal cancer. The risk of pharyngeal cancer for patients diagnosed with periodontitis within 3 years was 1.5-fold greater than in patients without periodontitis (adjusted odds ratio (aOR), 1.57; 95% confidence interval (CI), 1.17 to 2.10). Model 1 entailed conditional logistic regression with the covariates of history of periodontitis and total number of SRP incidents for the last 6 years, and the results showed that the aOR was 1.65 (95% CI, 1.24 to 2.20) in patients diagnosed with periodontitis over the last 3 years only, and the number of SRP incidents seems to be related to lower odds of pharyngeal cancer. Subjects undergoing more than 6 SRP treatments over the last 6 years had a significantly decreased OR of 0.70 (95% CI, 0.51 to 0.95) compared with individuals who had no SRP records, and the data show a lower OR in patients with at least 1 SRP (not all significant) within 6 years before the index date. Model 2 entailed inputting periodontitis history and the timing of SRP into conditional logistic regression analysis, and the results show that SRP intervention performed only during period 1 (over the past 3 years) had no significant protective effect. We found a decreased risk of pharyngeal cancer for patients who underwent SRP in period 2 (3 to 6 years before the index date), in whom the aOR was 0.75 (95% CI, 0.58 to 0.97), or in both period 1 and period 2 (aOR, 0.77; 95% CI, 0.61 to 0.96).

### 3.3. Effect of Periodontitis or SRP on the Risk on Anatomic Location of Cancer

The results of subset analysis based on anatomic location of cancer are shown in Table 3. For oropharyngeal cancer, newly diagnosed periodontitis within 3 years significantly increased the odds of oropharyngeal cancer (aOR, 2.22; 95% CI, 1.07 to 4.60). Yet, the risk of oropharyngeal cancer decreased roughly 50% for patients who had undergone SRP over the past 6 years. For hypopharyngeal cancer, the effect of periodontitis history or SRP was similar (but not as significant) to that for oropharyngeal cancer. However, nasopharyngeal cancer showed a nonsignificant relationship with periodontitis or SRP.

We further performed a comprehensive analysis of the risk of pharyngeal cancer, combining the two factors periodontitis and SRP (Table 4). When we selected a group with no periodontal disease and no SRP records as the reference group, we found the highest and a significantly increased risk of pharyngeal cancer for patients with newly diagnosed periodontitis within 3 years but who had not undergone SRP (aOR, 1.85; 95% CI, 1.07 to 3.19), followed by patients with newly observed periodontitis who had undergone SRP within 3 years (aOR, 1.47; 95% CI, 1.09 to 1.98). Moreover, we found a greater protective effect against pharyngeal cancer in patients with no periodontal disease but who had received regular SRP over the past 6 years (OR, 0.80; 95% CI, 0.43 to 1.49).

## 4. Discussion

In this population-based nested case–control study, we found a statistically significant higher risk of pharyngeal cancer in patients with periodontitis, with a 1.5-fold greater risk than those without periodontitis (aOR, 1.57; 95% CI, 1.17 to 2.10; Table 2). The higher risk was mainly attributed to the oropharyngeal cancer subgroup (aOR, 2.22; 95% CI, 1.07 to 4.60; Table 3). For patients with longer history of periodontitis more than 3 years, they had already undergone more times of dental visit, scaling and root planing and long-term follow up. The proportion of long-term scaling and root planing showed about 40% for more than 6 years (Figure 2.) compared with less than 10% of scaling and root planing for 3 years (−6 to −3 years from index date) in the group diagnosed periodontitis within 3 years, which can explain the higher OR in the former group. In addition, our study demonstrated that SRP exerts a potential protective effect that may reduce the risk of pharyngeal cancer, especially in the subgroup of patients with oropharyngeal cancer.

Oral cavity hygiene has been considered an essential factor related not only to periodontitis but also to a number of systemic diseases. Caused by bacterial accumulation of *Aggregatibacter actinomycetemcomitans*, *Porphyromonas gingivalis*, and *Tannerella forsythia* [15] or environmental factors such as aging, alcohol consumption, and cigarette smoking [16], the local inflammatory process and tissue destruction of the gums and alveolar bone release inflammatory factors, pathogens, and microbes into the circulatory system and result in systemic spread. Epstein–Barr virus and human papillomavirus, resulting in chronic periodontitis [17], aggressive periodontitis and carcinogenesis, are both well recognized major risk factors of oropharyngeal cancer [6,7]. Furthermore, poor oral hygiene with periodontal bacterial pathogen such as *Porphyromonas gingivalis* could also induce Epstein-Barr virus (EBV) reactivation [18,19]. Periodontitis has been confirmed to be a risk factor for systemic diseases [20,21], including cardiovascular diseases [22,23], respiratory diseases [24,25], musculoskeletal diseases [26,27]; adverse pregnancy outcomes [28,29], diabetes mellitus [30,31]; and even malignancy, particularly head and neck squamous cell carcinoma [32,33].

Most concern of the correlation of periodontitis and cancer is the biological plausibility, mentioned in a previous published review article. Even though previous data mostly derived from observational studies, these data and study outcomes meet temporality, strength, consistency and coherence of the Bradford Hill criteria, suggesting the positive correlation of periodontitis and head and neck cancer. Moreover, there are more current evidence and mechanism supporting the biological plausibility of periodontitis and head and neck cancer. Irani et al. integrated comprehensive data and mechanism including the contribution of microorganisms to carcinogenesis [34,35], possible signaling pathways involved in microbial carcinogenesis [36,37,38,39], involvement of inflammatory cells [40,41], cell proliferation, differentiation and angiogenesis caused by chronic inflammation, cytokines and chemokines in periodontitis and cancer development [42,43], and even the microRNAs [44,45,46,47] and salivary proteins as biomarkers [48]. This study concluded that inflammatory and sequential responses caused by periodontitis can increase the risk of genetic alterations and malignant transformation. Among the oral microorganisms, *P. gingivalis* and the activation of immunologic and inflammatory reactions has been frequency studied and considered as the underlying mechanism [49,50,51,52,53]. Zeng et al. also performed a meta-analysis of observational studies and confirmed that periodontitis is a significant risk factor for head and neck squamous cell carcinoma (OR, 2.63; 95% CI, 1.68 to 4.14; *p* < 0.001) [34]. Furthermore, in our study, the main anatomic location of cancer development was mainly in the oropharynx rather than the nasopharynx or hypopharynx in both groups with periodontitis for less and more than 3 years (periodontitis < 3 years: aOR [95% CI], 2.22 [1.07 to 4.60], 1.38 [0.96 to 1.98], 1.93 [0.92 to 4.05]; periodontitis ≥ 3 years: 1.52 [0.89 to 2.59], 1.09 [0.84 to 1.42], and 1.19 [0.73 to 1.96]).

The current treatment approach for pharyngeal cancer, based on the National Comprehensive Cancer Network Clinical Practice Guidelines [54], is composed of definitive radiotherapy or surgery for early-stage disease, surgery followed by adjuvant radiotherapy or chemoradiotherapy for locoregionally resectable advanced disease, and concomitant chemotherapy with radiotherapy for locoregionally nonresectable advanced disease and recurrent or metastatic disease [55]. The prognosis and treatment outcomes of patients depend on the stage and characteristics of their disease. Chiang et al. conducted a study using the Taiwan Cancer Registry database and showed 5-year age-standardized relative survival rates of 38.6% for oropharyngeal cancer and 28.2% for hypopharyngeal cancer [56]. Quality of life is an important issue as well. A systematic review focused on the health-related quality of life of patients with HNC who underwent treatment and identified a decline in health-related quality of life [57]. Both prevention with early detection and multidisciplinary care are required for better prognosis and long-term maintenance of quality of life.

SRP, which eradicates the inflammatory and carcinogenic factors by removing the dental plaque and calculus, is a nonsurgical treatment in the current clinical practice guideline for chronic periodontitis released by the Council on Scientific Affairs of the American Dental Association in 2015 [58]. Nevertheless, the correlation between SRP and pharyngeal cancer remains known. Current studies have mainly focused on posttreatment dental care in patients with HNC rather than disease prevention or early detection [59,60]. The results of our study demonstrated the protective effect of early diagnosis and early intervention of scaling and root planing for periodontitis on reduced risk of pharyngeal cancer, particularly in patients with a history of periodontitis longer than 3 years and early treatment with SRP (aOR, 0.87; 95% CI, 0.69 to 1.11). Patients with a periodontitis history less than 3 years include some individuals with late diagnosis of periodontitis, and their risk of pharyngeal cancer showed an obvious increase (aOR, 1.85 and 1.47; 95% CI, 1.07 to 3.19 and 1.09 to 1.98, respectively; Table 4). Regarding the results mentioned previously, the concept of oral hygiene and routine dental procedures as well as general health education should be increasingly advocated and promoted [61]. Even though National health insurance in Taiwan covers the costs of SRP, the utilization rate of dentist and oral health care remain low (lower than 50% in Taiwan). Since the high prevalence of periodontal disease and the risk of further oropharyngeal cancer, oral health care and dental procedure for oral hygiene such as SRP should be advocated for adequate utilization.

Our study demonstrated the protective effect of dental scaling and root planning toward oropharynx cancer. On the other hand, for head and neck cancer patients undergone oncology surgery, oral cavity reconstruction and radiotherapy, prosthetic rehabilitation had been proved as a vital component of treatment, not only restoring the oral function but also improving the quality of life. Beside oral hygiene maintenance for general population, multidisciplinary coordination of pre-treatment preparation, virtual surgical planning, treatment execution and oral rehabilitation for head and neck patient is also of utmost importance [62,63].

The strengths and novelties of our study are as follows. First, we used a nested case–control study design that possesses advantages of both a cohort study and a case–control study. Second, the subgroup analysis of anatomic location provided precise information regarding the risk of different cancers. Clinical physicians should pay more attention to early detection and management of oropharyngeal cancer in patients with chronic periodontitis. Third, we further explored the effect of early detection of periodontitis and early intervention of SRP on the risk of pharyngeal cancer. With the beneficial information provided in the study, modification and adjustment of health policy and clinical practice can be made.

There were also some limitations to this study. First, the NHIRD does not contain data on the severity and stage of periodontitis. This factor may have influenced the decisions regarding dental procedures for patients. Second, we did not have access to potential risk factors, including behavioral information related to smoking or alcohol and betel nut consumption, viral infection, and precancerous lesions. Third, other nonsurgical or surgical dental procedures were not analyzed in this study, which may be confounding factors. Therefore, further research is required to evaluate thoroughly dental procedures and their effect on the risk of pharyngeal cancer. Finally, lack of information on disease severity is one of the limitations for retrospective studies using the NHIRD. Current periodontitis stage and grading are based on American Academy of Periodontology guidelines announced in June 2018. Further well designed studies with stratification of stage and grade of periodontitis are needed for more detail information.

## 5. Conclusions

In summary, the subgroup analysis of this population-based nested case–control study revealed that periodontitis is associated with an increased risk of pharyngeal cancer, especially oropharyngeal cancer. Furthermore, SRP exhibited a protective effect with decreased risk of pharyngeal cancer in patients with periodontitis. Our results may draw more attention to oral hygiene in clinical practice and facilitate improvements in health policy.

## Figures and Tables

**Table 1:** Characteristics in case (patients with pharyngeal cancer) and control group.

Variables	Control	Pharyngeal Cancer	*p* Value
N	2584	1292	
Age at index date			0.927
<40	491 (19.00%)	246 (19.04%)	
40–59	1460 (56.50%)	740 (57.28%)	
60–79	581 (22.48%)	283 (21.90%)	
≥80	52 (2.01%)	23 (1.78%)	
Sex			0.656
Female	643 (24.88%)	330 (25.54%)	
Male	1941 (75.12%)	962 (74.46%)	
Urbanization			0.867
Urban	1415 (54.76%)	707 (54.72%)	
Sub-urban	829 (32.08%)	422 (32.66%)	
Rural	340 (13.16%)	163 (12.62%)	
Co-morbidities ^‡^			
Diabetes mellitus	356 (13.78%)	170 (13.16%)	0.596
Hypertension	689 (26.66%)	336 (26.01%)	0.662
Hyperlipidaemia	454 (17.57%)	215 (16.64%)	0.471
Chronic kidney disease	73 (2.83%)	44 (3.41%)	0.319
Depression	35 (1.35%)	20 (1.55%)	0.631
Stroke	106 (4.10%)	58 (4.49%)	0.573
Ulcer of esophagus	215 (8.32%)	107 (8.28%)	0.967
Allergies	850 (32.89%)	414 (32.04%)	0.594
COPD	342 (13.24%)	164 (12.69%)	0.637
Hepatitis	321 (12.42%)	169 (13.08%)	0.561
HPV	95(3.68%)	51(3.95%)	0.676
Amount of medical utilization ^†^			
Length of hospital stay (days)			0.772
0	2289 (88.58%)	1137 (88.00%)	
1–13	236 (9.13%)	121 (9.37%)	
≥14	59 (2.28%)	34 (2.63%)	
Time of dental visit			0.289
0	1592 (61.61%)	763 (59.06%)	
1–2	542 (20.98%)	284 (21.98%)	
≥3	450 (17.41%)	245 (18.96%)	

^‡^ Co-morbidity was identified within 2 years before index date. ^†^ Amount of medical utilization was identified within 1 year before index date.

**Table 2:** The management of periodontitis among pharyngeal cancer and control groups.

Variables	Control	Pharyngeal Cancer	OR for Model 1	OR for Model 2
History of periodontitis				
Non-periodontitis	939 (67.12%)	460 (32.88%)	Reference	Reference
<3 years	186 (58.13%)	134 (41.88%)	1.65 (1.24–2.20) *	1.57 (1.17–2.10) *
≥3 years	1459 (67.64%)	698 (32.36%)	1.20 (0.96–1.50)	1.18 (0.95–1.46)
Total amount of SRP within 6 years before index date				
0	897 (65.47%)	473 (34.53%)	Reference	
1	459 (65.38%)	243 (34.62%)	0.84 (0.66–1.06)	
2	338 (65.00%)	182 (35.00%)	0.87 (0.66–1.14)	
3	255 (67.64%)	122 (32.36%)	0.78 (0.57–1.05)	
4	196 (72.32%)	75 (27.68%)	0.63 (0.45–0.89) *	
5	139 (66.51%)	70 (33.49%)	0.83 (0.58–1.18)	
≥6	300 (70.26%)	127 (29.74%)	0.70 (0.51–0.95) *	
Utilization of dental scaling				
No use within 6 years	964 (65.49%)	508 (34.51%)		Reference
Only use in period 1	390 (62.9%)	230 (37.10%)		0.92 (0.72–1.17)
Only use in period 2	367 (69.64%)	160 (30.36%)		0.75 (0.58–0.97) *
Use in both periods	863 (68.66%)	394 (31.34%)		0.77 (0.61–0.96) *

OR, odds ratio was estimated by conditional logistic regression. Period 1: from index date to −3 years. Period 2: from −3 years to −6 years. * *p* value < 0.05.

**Table 3:** The management of periodontitis among pharyngeal cancer and control groups.

Variables	aOR (95% CI)		
	Oropharynx Cancer	Nasopharynx Cancer	Hypopharynx Cancer
History of periodontitis			
Non-periodontitis	Reference	Reference	Reference
<3 years	2.22 (1.07–4.60) *	1.38 (0.98–1.98)	1.93 (0.92–4.05)
≥3 years	1.52 (0.89–2.59)	1.09 (0.84–1.42)	1.19 (0.73–1.96)
Utilization of SRP			
No use within 6 years	Reference	Reference	Reference
Only use in period 1	0.57 (0.32–1.03)	1.29 (0.95–1.74)	0.43 (0.23–0.83) *
Only use in period 2	0.47 (0.25–0.90) *	0.96 (0.70–1.32)	0.58 (0.31–1.09)
Use in both periods	0.50 (0.28–0.89) *	1.13 (0.85–1.50)	0.26 (0.14–0.47) *

aOR, odds ratio was estimated by conditional logistic regression, and set the covariate of history for Periodontitis and Scaling and root planing in multiple regression. * *p* value < 0.05.

**Table 4:** Combination of periodontitis and dental scaling among pharyngeal cancer and control groups.

History for Periodontitis	Dental Scaling	Control	Pharyngeal Cancer	aOR
<3 years	No use	28 (51.85%)	26 (48.15%)	1.85 (1.07–3.19) *
	Only in period 1	126 (57.8%)	92 (42.20%)	1.47 (1.09–1.98) *
	Only in period 2	3 (100.00%)	0 (0.00%)	
	In both periods	29 (64.44%)	16 (35.56%)	1.12 (0.60–2.09)
≥3 years	No use	148 (61.41%)	93 (38.59%)	1.29 (0.96–1.72)
	Only in period 1	199 (65.03%)	107 (34.97%)	1.09 (0.84–1.42)
	Only in period 2	315 (70.00%)	135 (30.00%)	0.87 (0.69–1.11)
	In both periods	797 (68.71%)	363 (31.29%)	0.92 (0.78–1.10)
Non-periodontitis	No use	788 (66.95%)	389 (33.05%)	Reference
	Only in period 1	65 (67.71%)	31 (32.29%)	0.96 (0.62–1.49)
	Only in period 2	49 (66.22%)	25 (33.78%)	1.02 (0.62–1.69)
	In both periods	37 (71.15%)	15 (28.85%)	0.80 (0.43–1.49)

aOR, odds ratio was estimated by conditional logistic regression, and set the covariate of History for periodontitis and Scaling and root planing in multiple regression. Period 1: From index date to −3 years. Period 2: From −3 years to −6 years. * *p* value < 0.05.
